# Erratum to Intended, Unintended, Unanticipated? Consequences of Social Distancing Measures for Nursing Home Residents During the Covid-19 Pandemic

**DOI:** 10.1177/23333936231186016

**Published:** 2023-07-07

Tingvold, L., Moholt, J-M., Førland, O., Jacobsen, FF. & Tranevåg, O. (2023). Intended, Unintended, Unanticipated? Consequences of Social Distancing Measures for Nursing Home Residents During the Covid-19 Pandemic. *Global Qualitative Nursing Research, 10*, 1-15. DOI:10.1177/23333936231176204

The below corrections have been made post publication of the article:

1. The correct spelling of author’s name is Oscar Tranvåg (not Tranevåg). It has been corrected online.
2. The Results heading has been inserted before the heading Confusion and Communication Challenges.

